# Korobov polynomials of the third kind and of the fourth kind

**DOI:** 10.1186/s40064-015-1405-9

**Published:** 2015-10-14

**Authors:** Taekyun Kim, Dae San Kim

**Affiliations:** Department of Mathematics, College of Science, Tianjin Polytechnic University, Tianjin, 300387 China; Department of Mathematics, Kwangwoon University, Seoul, 139-701 Republic of Korea; Department of Mathematics, Sogang University, Seoul, 121-742 Republic of Korea

**Keywords:** Korobov polynomials of the third kind and of the fourth kind, umbral calculus, 05A19, 05A40, 11B83

## Abstract

The first degenerate version of the Bernoulli polynomials of the second kind appeared in the paper by Korobov (Math Notes 2:77–19, 1996; Proceedings of the IV international conference modern problems of number theory and its applications, pp 40–49, 2001). In this paper, we study two degenerate versions of the Bernoulli polynomials of the second kind which will be called Korobov polynomials of third kind and of the fourth kind. Some properties, identities, recurrence relations and connections with other polynomials are investigated by using umbral calculus.

## Background

The Bernoulli polynomials of the second kind $b_{n}\left( x\right)$ are given by the generating function

1.1 $$\begin{aligned} \frac{t}{\log \left( 1+t\right) }\left( 1+t\right) ^{x}=\sum _{n=0}^{\infty }b_{n}\left( x\right) \frac{t^{n}}{n!},\quad \left( \text {see Kim et al. 2014, 2015; Roman 1984}\right) . \end{aligned}$$

When $x=0$, $b_{n}=b_{n}\left( 0\right)$ are called Bernoulli numbers of the second kind. The degenerate version of the Bernoulli polynomials of the second kind are called Korobov polynomials of the first kind. We note here that the Carlitz degerate Bernoulli polynomials were rediscovered by Ustinov under the name of Korobov polynomial of the second kind (see Pylypiv and Maliarchuk 2014; Ustinov 2003).

The Daehee polynomials $D_{n}\left( x\right)$ are defined by the generating function

1.2 $$\begin{aligned} \frac{\log \left( 1+t\right) }{t}\left( 1+t\right) ^{x}=\sum _{n=0}^{\infty }D_{n}\left( x\right) \frac{t^{n}}{n!},\quad \left( \text {see Kim et al. 2014, 2015}\right) . \end{aligned}$$

For $x=0$, $D_{n}=D_{n}\left( 0\right)$ are called Daehee numbers.

The Korobov polynomials $K_{n}\left( \lambda ,x\right)$ of the first kind are given by the generating function

1.3 $$\begin{aligned} \frac{\lambda t}{\left( 1+t\right) ^{\lambda }-1}\left( 1+t\right) ^{x}=\sum _{n=0}^{\infty }K_{n}\left( x\mid \lambda \right) \frac{t^{n}}{n!},\quad \left( \text {see Korobov 1996; Korobov 2001}\right) . \end{aligned}$$

When $x=0$, $K_{n}\left( \lambda \right) =K_{n}\left( 0\mid \lambda \right)$ are called Korobov numbers of the first kind.

In the following, we will review very briefly some necessary things on umbral calculus. Our basic reference is Roman (1984). Also, one is asked to look at more recent papers on umbral calculus (Nisar et al. 2015; Srivastava et al. 2014).

Let $\mathbb {C}$ be the complex number field and let $\mathcal {F}$ be the set of all formal power series in the variable *t* over $\mathbb {C}$ with

1.4 $$\begin{aligned} \mathcal {F}=\left\{ \left. f\left( t\right) =\sum _{k=0}^{\infty }\frac{a_{k}}{k!}t^{k}\right| a_{k}\in \mathbb {C}\right\} . \end{aligned}$$

Let $\mathbb {P}=\mathbb {C}\left[ x\right]$ and let $\mathbb {P}^{*}$ be the vector space of all linear functionals on $\mathbb {P}$. For $L\in \mathbb {P}^{*}$, the action of the linear functional *L* on a polynomial $p\left( x\right)$ is denoted by $\left\langle \left. L\right| p\left( x\right) \right\rangle$ with

$$\begin{aligned} \left\langle \left. L+M\right| p\left( x\right) \right\rangle =\left\langle \left. L\right| p\left( x\right) \right\rangle +\left\langle \left. M\right| p\left( x\right) \right\rangle ,\quad \left\langle \left. cL\right| p\left( x\right) \right\rangle =c\left\langle \left. L\right| p\left( x\right) \right\rangle , \end{aligned}$$

where *c* is a complex constant (see Kim 2014; Roman 1984).

For $f\left( t\right) =\sum _{k=0}^{\infty }a_{k}\frac{t^{k}}{k!}\in \mathcal {F}$, we define a linear functional on $\mathbb {P}$ by setting

1.5 $$\begin{aligned} \left\langle \left. f\left( t\right) \right| x^{n}\right\rangle =a_{n},\quad \text {for all }n\ge 0,\quad \left( \text {see Kim et al. 2014; Roman 1984}\right) . \end{aligned}$$

Thus, by (1.5), we easily get

1.6 $$\begin{aligned} \left\langle \left. t^{k}\right| x^{n}\right\rangle =n!\delta _{n,k},\quad \left( n,k\ge 0\right) , \end{aligned}$$

where $\delta _{n,k}$ is the Kronecker’s symbol.

Let $f_{L}\left( t\right) =\sum _{k=0}^{\infty }\left\langle \left. L\right| x^{k}\right\rangle \frac{t^{n}}{k!}$. Then, by (1.6), we get $\left\langle \left. f_{L}\left( t\right) \right| x^{n}\right\rangle =\left\langle \left. L\right| x^{n}\right\rangle$. Additionallly, the mapping $L\mapsto f_{L}\left( t\right)$ is a vector space isomorphism from $\mathbb {P}^{*}$ onto $\mathcal {F}$. Henceforth, $\mathcal {F}$ denotes both the algebra of formal power series in *t* and the vector space of all linear functionals on $\mathbb {P}$, and so an element $f\left( t\right)$ of $\mathcal {F}$ can be regarded as both a formal power series and a linear functional. We refer to $\mathcal {F}$ as the umbral algebra. The umbral calculus is the study of umbral algebra (see Kim 2014; Roman 1984). From (1.6), we can easily derive $\left\langle \left. e^{yt}\right| x^{n}\right\rangle =y^{n}$. So $\left\langle \left. e^{yt}\right| p\left( x\right) \right\rangle =p\left( y\right)$. The order $o\left( f\left( t\right) \right)$ of a power series $f\left( t\right) \left( \ne 0\right)$ is the smallest nonnegative integer *k* for which the coefficient at $t^{k}$ does not vanish. For $f\left( t\right) \in \mathcal {F}$ and $p\left( x\right) \in \mathbb {P}$, we have

1.7 $$\begin{aligned} f\left( t\right) =\sum _{k=0}^{\infty }\left\langle \left. f\left( t\right) \right| x^{k}\right\rangle \frac{t^{k}}{k!},\quad p\left( x\right) =\sum _{k=0}^{\infty }\left\langle \left. t^{k}\right| p\left( x\right) \right\rangle \frac{x^{k}}{k!}. \end{aligned}$$

Thus, by (1.7), we get

1.8 $$\begin{aligned} p^{\left( k\right) }\left( 0\right) =\left. \left( \frac{d}{dx}\right) ^{k}p\left( x\right) \right| _{x=0}=\left\langle \left. t^{k}\right| p\left( x\right) \right\rangle =\left\langle \left. 1\right| p^{\left( k\right) }\left( x\right) \right\rangle . \end{aligned}$$

From (1.8), we note that

1.9 $$\begin{aligned} t^{k}p\left( x\right) =p^{\left( k\right) }\left( x\right) =\frac{d^{k}}{dx^{k}}p\left( x\right) ,\quad e^{yt}p\left( x\right) =p\left( x+y\right) ,\quad \left( \text {see Roman 1984}\right) . \end{aligned}$$

Let $$f\left( t\right) ,g\left( t\right) \in \mathcal {F}$$ such that $$o\left( f\left( t\right) \right) =1$$ and $$o\left( g\left( t\right) \right) =0$$. Then there exists a unique sequence $$s_{n}\left( x\right)$$$$\left( \deg s_{n}\left( x\right) =n\right)$$ of polynomials such that $$\left\langle \left. g\left( t\right) f\left( t\right) ^{k}\right| s_{n}\left( x\right) \right\rangle$$$$=n!\delta _{n,k}$$, for $$n,k\ge 0$$. The sequence $$s_{n}\left( x\right)$$ is called the Sheffer sequence for the pair $$\left( g\left( t\right) ,f\left( t\right) \right)$$, which is denoted by $$s_{n}\left( x\right) \sim \left( g\left( t\right) ,f\left( t\right) \right)$$. For $$s_{n}\left( x\right) \sim \left( g\left( t\right) ,f\left( t\right) \right) ,$$ we have

1.10 $$\begin{aligned} f\left( t\right) s_{n}\left( x\right) =ns_{n-1}\left( x\right) ,\quad \left( n\in \mathbb {N}\cup \left\{ 0\right\} \right) , \end{aligned}$$

and

1.11 $$\begin{aligned} \frac{1}{g\left( \overline{f}\left( t\right) \right) }e^{x\overline{f}\left( t\right) }=\sum _{k=0}^{\infty }\frac{s_{k}\left( x\right) }{k!}t^{k},\quad \text {for all }x\in \mathbb {C}. \end{aligned}$$

Here $\overline{f}\left( t\right)$ is the compositional inverse of $f\left( t\right)$ (see Kim and Mansour 2014; Roman 1984).

The conjugation representation for $s_{n}\left( x\right) \sim \left( g\left( t\right) ,f\left( t\right) \right)$ is given by

1.12 $$\begin{aligned} s_{n}\left( x\right) =\sum _{k=0}^{n}\frac{1}{k!}\left\langle \left. g\left( \overline{f}\left( t\right) \right) ^{-1}\overline{f}\left( t\right) ^{k}\right| x^{n}\right\rangle x^{k},\quad \left( n\ge 0\right) ,\quad \left( \text {see Carlitz 1979; Roman 1984}\right) . \end{aligned}$$

Let us consider the following two Sheffer sequences:

1.13 $$\begin{aligned} s_{n}\left( x\right) \sim \left( g\left( t\right) ,f\left( t\right) \right) ,\quad r_{n}\left( x\right) \sim \left( h\left( t\right) ,l\left( t\right) \right) . \end{aligned}$$

Then, we have

1.14 $$\begin{aligned} s_{n}\left( x\right) =\sum _{m=0}^{n}C_{n,m}r_{m}\left( x\right) ,\quad \left( n\ge 0\right) , \end{aligned}$$

where

1.15 $$\begin{aligned} C_{n,m}=\frac{1}{m!}\left\langle \left. \frac{h\left( \overline{f}\left( t\right) \right) }{g\left( \overline{f}\left( t\right) \right) }\left( l\left( \overline{f}\left( t\right) \right) \right) ^{m}\right| x^{n}\right\rangle ,\quad \left( \text {see Kim et al. 2014; Roman 1984}\right) . \end{aligned}$$

The first degenerate version of the Bernoulli polynomials of the second kind appeared in the paper by Korobov (2001; 1996). In this paper, we study two degenerate versions of the Bernoulli polynomials of the second kind which will be called Korobov polynomials of the third kind and of the fourth kind. Some properties, identities and recurrence relations for them are investigated by using umbral calculus. In addition, some connections with other polynomials are studied for which one refers to the related papers (Dattoli et al. 2006, 2004).

## Korobov polynomials of the third kind and of the fourth kind

Now, we introduce Korobov polynomials of the third kind $K_{n,3}\left( x\mid \lambda \right)$ and of the fourth kind $K_{n,4}\left( x\mid \lambda \right)$, respectively, given by the generating functions

2.1 $$\begin{aligned} \frac{\log \left( 1+\lambda t\right) }{\lambda \log \left( 1+t\right) }\left( 1+t\right) ^{x}=\sum _{n=0}^{\infty }K_{n,3}\left( x\mid \lambda \right) \frac{t^{n}}{n!}, \end{aligned}$$

and

2.2 $$\begin{aligned} \frac{\log \left( 1+\lambda t\right) }{\left( 1+t\right) ^{\lambda }-1}\left( 1+t\right) ^{x}=\sum _{n=0}^{\infty }K_{n,4}\left( x\mid \lambda \right) \frac{t^{n}}{n!}. \end{aligned}$$

When $x=0$, $K_{n,3}\left( \lambda \right) =K_{n,3}\left( 0,\lambda \right)$ and $K_{n,4}\left( \lambda \right) =K_{n,4}\left( 0\mid \lambda \right)$ are called Korobov numbers of the third kind and of the fourth kind, respectively.

As all $\frac{\lambda t}{\left( 1+t\right) ^{\lambda }-1}=\frac{t}{\frac{\left( 1+t\right) ^{\lambda }-1}{\lambda }}$, $\frac{\log \left( 1+\lambda t\right) }{\lambda \log \left( 1+t\right) }=\frac{\log \left( 1+\lambda t\right) ^{\frac{1}{\lambda }}}{\log \left( 1+t\right) }$, $\frac{\log \left( 1+\lambda t\right) }{\left( 1+t\right) ^{\lambda }-1}=\frac{\log \left( 1+\lambda t\right) ^{\frac{1}{\lambda }}}{\frac{\left( 1+\lambda t\right) ^{\lambda }-1}{\lambda }}$ tend to $\frac{t}{\log \left( 1+t\right) }$ as $\lambda \rightarrow 0$, $\lim _{\lambda \rightarrow 0}K_{n}\left( x\mid \lambda \right) =\lim _{\lambda \rightarrow 0}K_{n,3}\left( x\mid \lambda \right) =\lim _{\lambda \rightarrow 0}K_{n,4}\left( x\mid \lambda \right) =b_{n}\left( x\right)$, $\left( n\ge 0\right)$. We observe first that $K_{n,3}\left( x\mid \lambda \right)$ and $K_{n,4}\left( x\mid \lambda \right)$ are Sheffer sequences for the respective pairs $\left( \frac{\lambda t}{\log \left( 1+\lambda \left( e^{t}-1\right) \right) },e^{t}-1\right)$ and $\left( \frac{e^{\lambda t}-1}{\log \left( 1+\lambda \left( e^{t}-1\right) \right) },e^{t}-1\right)$. That is,

$$\begin{aligned} K_{n,3}\left( x\mid \lambda \right) \sim \left( \frac{\lambda t}{\log \left( 1+\lambda \left( e^{t}-1\right) \right) },e^{t}-1\right) , \end{aligned}$$

and

2.3 $$\begin{aligned} K_{n,4}\left( x\mid \lambda \right) \sim \left( \frac{e^{\lambda t}-1}{\log \left( 1+\lambda \left( e^{t}-1\right) \right) },e^{t}-1\right) . \end{aligned}$$

From (1.12) and (2.2), we have

2.4 $$\begin{aligned} K_{n,3}\left( x\mid \lambda \right) =\sum _{k=0}^{n}\frac{1}{k!}\left\langle \left. \frac{\log \left( 1+\lambda t\right) }{\lambda \log \left( 1+t\right) }\left( \log \left( 1+t\right) \right) ^{k}\right| x^{n}\right\rangle x^{k}. \end{aligned}$$

We observe that

2.5 $$\begin{aligned}&\left\langle \left. \frac{\log \left( 1+\lambda t\right) }{\lambda \log \left( 1+t\right) }\left( \log \left( 1+x\right) \right) ^{k}\right| x^{n}\right\rangle \nonumber \\&=\left\langle \left. \frac{\log \left( 1+\lambda t\right) }{\lambda \log \left( 1+t\right) }\right| \left( \log \left( 1+x\right) \right) ^{k}x^{n}\right\rangle \nonumber \\&=\left\langle \left. \frac{\log \left( 1+\lambda t\right) }{\lambda \log \left( 1+t\right) }\right| k!\sum _{l=k}^{\infty }S_{1}\left( l,k\right) \frac{t^{l}}{l!}x^{n}\right\rangle \nonumber \\&=k!\sum _{l=k}^{n}\left( {\begin{array}{c}n\\ l\end{array}}\right) S_{1}\left( l,k\right) \left\langle \left. \frac{\log \left( 1+\lambda t\right) }{\lambda t}\right| \frac{t}{\log \left( 1+t\right) }x^{n-l}\right\rangle \nonumber \\&=k!\sum _{l=k}^{n}\left( {\begin{array}{c}n\\ l\end{array}}\right) S_{1}\left( l,k\right) \left\langle \left. \frac{\log \left( 1+\lambda t\right) }{\lambda t}\right| \sum _{m=0}^{\infty }b_{m}\frac{t^{m}}{m!}x^{n-l}\right\rangle \nonumber \\&=k!\sum _{l=k}^{n}\left( {\begin{array}{c}n\\ l\end{array}}\right) S_{1}\left( l,k\right) \sum _{m=0}^{n-l}\left( {\begin{array}{c}n-l\\ m\end{array}}\right) b_{m}\left\langle \left. \frac{\log \left( 1+\lambda t\right) }{\lambda t}\right| x^{n-l-m}\right\rangle \nonumber \\&=k!\sum _{l=k}^{n}\left( {\begin{array}{c}n\\ l\end{array}}\right) S_{1}\left( l,k\right) \sum _{m=0}^{n-l}\left( {\begin{array}{c}n-l\\ m\end{array}}\right) b_{m}\left\langle \left. \sum _{j=0}^{\infty }D_{j}\lambda ^{j}\frac{t^{j}}{j!}\right| x^{n-l-m}\right\rangle \nonumber \\&=k!\sum _{l=k}^{n}\left( {\begin{array}{c}n\\ l\end{array}}\right) S_{1}\left( l,k\right) \sum _{m=0}^{n-l}\left( {\begin{array}{c}n-l\\ m\end{array}}\right) b_{m}D_{n-l-m}\lambda ^{n-l-m}\nonumber \\&=k!\sum _{l=k}^{n}\sum _{m=0}^{n-l}\left( {\begin{array}{c}n\\ l\end{array}}\right) \left( {\begin{array}{c}n-l\\ m\end{array}}\right) S_{1}\left( l,k\right) b_{m}D_{n-l-m}\lambda ^{n-l-m}, \end{aligned}$$

where $S_{1}\left( n,m\right)$ is the Stirling number of the first kind defined by

$$\begin{aligned} x\left( x-1\right) \ldots \left( x-n+1\right) =\left( x\right) _{n}=\sum _{l=0}^{n}S_{1}\left( n,l\right) x^{l},\quad \left( n\ge 0\right) . \end{aligned}$$

Therefore, by (2.4) and (2.5), we have

### **Theorem 1**

*For*$n\ge 0$*, we have*

$$\begin{aligned} K_{n,3}\left( x\mid \lambda \right) =\sum _{k=0}^{n}\left( \sum _{l=k}^{n}\sum _{m=0}^{n-l}\left( {\begin{array}{c}n\\ l\end{array}}\right) \left( {\begin{array}{c}n-l\\ m\end{array}}\right) S_{1}\left( l,k\right) b_{n-l-m}D_{m}\lambda ^{m}\right) x^{k}. \end{aligned}$$

From (1.12) and (2.3), we have

2.6 $$\begin{aligned} K_{n,4}\left( x\mid \lambda \right) =\sum _{k=0}^{n}\frac{1}{k!}\left\langle \left. \frac{\log \left( 1+\lambda t\right) }{\left( 1+t\right) ^{\lambda }-1}\left( \log \left( 1+t\right) \right) ^{k}\right| x^{n}\right\rangle x^{k}. \end{aligned}$$

We observe that

2.7 $$\begin{aligned}&\left\langle \left. \frac{\log \left( 1+\lambda t\right) }{\left( 1+t\right) ^{\lambda }-1}\left( \log \left( 1+t\right) \right) ^{k}\right| x^{n}\right\rangle \nonumber \\&=k!\sum _{l=k}^{n}\left( {\begin{array}{c}n\\ l\end{array}}\right) S_{1}\left( l,k\right) \left\langle \left. \frac{\log \left( 1+\lambda t\right) }{\lambda t}\frac{\lambda t}{\left( 1+t\right) ^{\lambda }-1}\right| x^{n-l}\right\rangle \nonumber \\&=k!\sum _{l=k}^{n}\left( {\begin{array}{c}n\\ l\end{array}}\right) S_{1}\left( l,k\right) \left\langle \left. \frac{\log \left( 1+\lambda t\right) }{\lambda t}\right| \sum _{m=0}^{\infty }K_{m}\left( \lambda \right) \frac{t^{m}}{m!}x^{n-l}\right\rangle \nonumber \\&=k!\sum _{l=k}^{n}\left( {\begin{array}{c}n\\ l\end{array}}\right) S_{1}\left( l,k\right) \sum _{m=0}^{n-l}\left( {\begin{array}{c}n-l\\ m\end{array}}\right) K_{m}\left( \lambda \right) \left\langle \left. \frac{\log \left( 1+\lambda t\right) }{\lambda t}\right| x^{n-l-m}\right\rangle \nonumber \\&=k!\sum _{l=k}^{n}\left( {\begin{array}{c}n\\ l\end{array}}\right) S_{1}\left( l,k\right) \sum _{m=0}^{n-l}\left( {\begin{array}{c}n-l\\ m\end{array}}\right) K_{m}\left( \lambda \right) D_{n-l-m}\lambda ^{n-l-m}\nonumber \\&=k!\sum _{l=k}^{n}\sum _{m=0}^{n-l}\left( {\begin{array}{c}n\\ l\end{array}}\right) \left( {\begin{array}{c}n-l\\ m\end{array}}\right) S_{1}\left( l,k\right) K_{n-l-m}\left( \lambda \right) D_{m}\lambda ^{m}. \end{aligned}$$

Therefore, by (2.6) and (2.7), we obtain the following theorem.

### **Theorem 2**

*For*$n\ge 0$*, we have*

$$\begin{aligned} K_{n,4}\left( x\mid \lambda \right) =\sum _{k=0}^{n}\left( \sum _{l=k}^{n}\sum _{m=0}^{n-l}\left( {\begin{array}{c}n\\ l\end{array}}\right) \left( {\begin{array}{c}n-l\\ m\end{array}}\right) S_{1}\left( l,k\right) K_{n-l-m}\left( \lambda \right) D_{m}\lambda ^{m}\right) x^{k}. \end{aligned}$$

By (1.6) and (2.1), we easily get

2.8 $$\begin{aligned} K_{n,3}\left( y\mid \lambda \right)&=\left\langle \left. \frac{\log \left( 1+\lambda t\right) }{\lambda \log \left( 1+t\right) }\left( 1+t\right) ^{y}\right| x^{n}\right\rangle \nonumber \\&=\left\langle \left. \frac{\log \left( 1+\lambda t\right) }{\lambda \log \left( 1+t\right) }\right| \left( 1+t\right) ^{y}x^{n}\right\rangle \nonumber \\&=\left\langle \left. \frac{\log \left( 1+\lambda t\right) }{\lambda \log \left( 1+t\right) }\right| \sum _{l=0}^{\infty }\left( y\right) _{l}\frac{t^{l}}{l!}x^{n}\right\rangle \nonumber \\&=\sum _{l=0}^{n}\left( {\begin{array}{c}n\\ l\end{array}}\right) \left( y\right) _{l}\left\langle \left. \frac{\log \left( 1+\lambda t\right) }{\lambda \log \left( 1+t\right) }\right| x^{n-l}\right\rangle \nonumber \\&=\sum _{l=0}^{n}\left( {\begin{array}{c}n\\ l\end{array}}\right) \left( y\right) _{l}\sum _{m=0}^{n-l}\left( {\begin{array}{c}n-l\\ m\end{array}}\right) b_{m}D_{n-l-m}\lambda ^{n-l-m}\nonumber \\&=\sum _{l=0}^{n}\left( {\begin{array}{c}n\\ l\end{array}}\right) \left( y\right) _{l}\sum _{m=0}^{n-l}\left( {\begin{array}{c}n-l\\ m\end{array}}\right) b_{n-l-m}D_{m}\lambda ^{m}\nonumber \\&=\sum _{l=0}^{n}\left( \sum _{m=0}^{n-l}\left( {\begin{array}{c}n\\ l\end{array}}\right) \left( {\begin{array}{c}n-l\\ m\end{array}}\right) b_{n-l-m}D_{m}\lambda ^{m}\right) \left( y\right) _{l}. \end{aligned}$$

Thus, by (2.8), we get

2.9 $$\begin{aligned} K_{n,3}\left( x\mid \lambda \right) =\sum _{l=0}^{n}\left( \sum _{m=0}^{n-l}\left( {\begin{array}{c}n\\ l\end{array}}\right) \left( {\begin{array}{c}n-l\\ m\end{array}}\right) b_{n-l-m}D_{m}\lambda ^{m}\right) \left( x\right) _{l},\quad \left( n\ge 0\right) . \end{aligned}$$

From (1.6) and (2.2), we note that

2.10 $$\begin{aligned} K_{n,4}\left( y\mid \lambda \right)&=\left\langle \left. \frac{\log \left( 1+\lambda t\right) }{\left( 1+t\right) ^{\lambda }-1}\left( 1+t\right) ^{y}\right| x^{n}\right\rangle \nonumber \\&=\left\langle \left. \frac{\log \left( 1+\lambda t\right) }{\left( 1+t\right) ^{\lambda }-1}\right| \left( 1+t\right) ^{y}x^{n}\right\rangle \nonumber \\&=\sum _{l=0}^{n}\left( {\begin{array}{c}n\\ l\end{array}}\right) \left( y\right) _{l}\left\langle \left. \frac{\log \left( 1+\lambda t\right) }{\left( 1+t\right) ^{\lambda }-1}\right| x^{n-l}\right\rangle \nonumber \\&=\sum _{l=0}^{n}\left( {\begin{array}{c}n\\ l\end{array}}\right) \left( y\right) _{l}\sum _{m=0}^{n-l}\left( {\begin{array}{c}n-l\\ m\end{array}}\right) K_{m}\left( \lambda \right) D_{n-l-m}\lambda ^{n-l-m}\nonumber \\&=\sum _{l=0}^{n}\left( {\begin{array}{c}n\\ l\end{array}}\right) \left( y\right) _{l}\sum _{m=0}^{n-l}\left( {\begin{array}{c}n-l\\ m\end{array}}\right) K_{n-l-m}\left( \lambda \right) D_{m}\lambda ^{m}\nonumber \\&=\sum _{l=0}^{n}\left( \sum _{m=0}^{n-l}\left( {\begin{array}{c}n\\ l\end{array}}\right) \left( {\begin{array}{c}n-l\\ m\end{array}}\right) K_{n-l-m}\left( \lambda \right) D_{m}\lambda ^{m}\right) \left( y\right) _{l}. \end{aligned}$$

Thus, by (2.10), we get

2.11 $$\begin{aligned} K_{n,4}\left( x\mid \lambda \right) =\sum _{l=0}^{n}\left( \sum _{m=0}^{n-l}\left( {\begin{array}{c}n\\ l\end{array}}\right) \left( {\begin{array}{c}n-l\\ m\end{array}}\right) K_{n-l-m}\left( \lambda \right) D_{m}\lambda ^{m}\right) \left( x\right) _{l}. \end{aligned}$$

From (2.2), we note that

2.12 $$\begin{aligned}&K_{n,3}\left( x\mid \lambda \right) \sim \left( \frac{\lambda t}{\log \left( 1+\lambda \left( e^{t}-1\right) \right) },e^{t}-1\right) \nonumber \\ \iff&\frac{\lambda t}{\log \left( 1+\lambda \left( e^{t}-1\right) \right) }K_{n,3}\left( x\mid \lambda \right) =\left( x\right) _{n}\sim \left( 1,e^{t}-1\right) . \end{aligned}$$

By (2.12), we get

2.13 $$\begin{aligned} K_{n,3}\left( x\mid \lambda \right)&=\frac{\log \left( 1+\lambda \left( e^{t}-1\right) \right) }{\lambda t}\left( x\right) _{n}\nonumber \\&=\sum _{k=0}^{n}S_{1}\left( n,k\right) \frac{\log \left( 1+\lambda \left( e^{t}-1\right) \right) }{\lambda t}x^{k}\nonumber \\&=\sum _{k=0}^{n}S_{1}\left( n,k\right) \frac{e^{t}-1}{t}\frac{\log \left( 1+\lambda \left( e^{t}-1\right) \right) }{\lambda \left( e^{t}-1\right) }x^{k}\nonumber \\&=\sum _{k=0}^{n}S_{1}\left( n,k\right) \frac{e^{t}-1}{t}\sum _{l=0}^{k}D_{l}\lambda ^{l}\frac{\left( e^{t}-1\right) ^{l}}{l!}x^{k}\nonumber \\&=\sum _{k=0}^{n}S_{1}\left( n,k\right) \frac{e^{t}-1}{t}\sum _{l=0}^{k}D_{l}\lambda ^{l}\sum _{m=l}^{\infty }S_{2}\left( m,l\right) \frac{t^{m}}{m!}x^{k}\nonumber \\&=\sum _{k=0}^{n}S_{1}\left( n,k\right) \sum _{l=0}^{k}D_{l}\lambda ^{l}\sum _{m=l}^{k}\left( {\begin{array}{c}k\\ m\end{array}}\right) S_{2}\left( m,l\right) \frac{e^{t}-1}{t}x^{k-m}. \end{aligned}$$

We observe that

2.14 $$\begin{aligned} \frac{e^{t}-1}{t}x^{k-m}&=\sum _{j=1}^{\infty }\frac{t^{j-1}}{j!}x^{k-m}\nonumber \\&=\sum _{j=0}^{\infty }\frac{1}{\left( j+1\right) !}t^{j}x^{k-m}\nonumber \\&=\sum _{j=0}^{k-m}\frac{1}{\left( j+1\right) !}t^{j}x^{k-m}\nonumber \\&=\sum _{j=0}^{k-m}\frac{1}{j+1}\left( {\begin{array}{c}k-m\\ j\end{array}}\right) x^{k-m-j}\nonumber \\&=\sum _{j=0}^{k-m}\frac{1}{k-m-j+1}\left( {\begin{array}{c}k-m\\ j\end{array}}\right) x^{j}. \end{aligned}$$

Thus, by (2.13) and (2.14), we have

2.15 $$\begin{aligned}&K_{n,3}\left( x\mid \lambda \right) \nonumber \\&=\sum _{k=0}^{n}\sum _{l=0}^{k}\sum _{m=l}^{k}\sum _{j=0}^{k-m}\frac{1}{k-m-j+1}\left( {\begin{array}{c}k\\ m\end{array}}\right) \left( {\begin{array}{c}k-m\\ j\end{array}}\right) S_{1}\left( n,k\right) S_{2}\left( m,l\right) D_{l}\lambda ^{l}x^{j}\nonumber \\&=\sum _{k=0}^{n}\sum _{l=0}^{k}\sum _{m=0}^{k-l}\sum _{j=0}^{m}\frac{1}{m-j+1}\left( {\begin{array}{c}k\\ m\end{array}}\right) \left( {\begin{array}{c}m\\ j\end{array}}\right) S_{1}\left( n,k\right) S_{2}\left( k-m,l\right) D_{l}\lambda ^{l}x^{j}\nonumber \\&=\sum _{j=0}^{n}\left( \sum _{k=j}^{n}\sum _{l=0}^{k-j}\sum _{m=j}^{k-l}\frac{1}{k+1}\left( {\begin{array}{c}k+1\\ m+1\end{array}}\right) \left( {\begin{array}{c}m+1\\ j\end{array}}\right) S_{1}\left( n,k\right) S_{2}\left( k-m,l\right) D_{l}\lambda ^{l}\right) x^{j}, \end{aligned}$$

where $S_{2}\left( n,k\right)$ is the Stirling number of the second kind given by

$$\begin{aligned} x^{n}=\sum _{l=0}^{n}S_{2}\left( n,l\right) \left( x\right) _{l},\quad \left( n\ge 0\right) . \end{aligned}$$

Therefore, by (2.15), we obtain the following theorem expressing $K_{n,3}\left( x\mid \lambda \right)$ in terms of the Stirling numbers of the first kind and of the second and Daehee numbers.

### **Theorem 3**

*For*$n\ge 0$*, we have*

$$\begin{aligned}&K_{n,3}\left( x\mid \lambda \right) \\&=\sum _{j=0}^{n}\left( \sum _{k=j}^{n}\sum _{l=0}^{k-j}\sum _{m=j}^{k-l}\frac{1}{k+1}\left( {\begin{array}{c}k+1\\ m+1\end{array}}\right) \left( {\begin{array}{c}m+1\\ j\end{array}}\right) S_{1}\left( n,k\right) S_{2}\left( k-m,l\right) D_{l}\lambda ^{l}\right) x^{j}. \end{aligned}$$

From (2.3), we have

2.16 $$\begin{aligned} \frac{e^{\lambda t}-1}{\log \left( 1+\lambda \left( e^{t}-1\right) \right) }K_{n,4}\left( x\mid \lambda \right) =\left( x\right) _{n}\sim \left( 1,e^{t}-1\right) ,\quad \left( n\ge 0\right) . \end{aligned}$$

Thus, by (2.16), we get

2.17 $$\begin{aligned} K_{n,4}\left( x\mid \lambda \right)&=\frac{\log \left( 1+\lambda \left( e^{t}-1\right) \right) }{e^{\lambda t}-1}\left( x\right) _{n}\nonumber \\&=\sum _{k=0}^{n}S_{1}\left( n,k\right) \frac{\log \left( 1+\lambda \left( e^{t}-1\right) \right) }{e^{\lambda t}-1}x^{k}\nonumber \\&=\sum _{k=0}^{n}S_{1}\left( n,k\right) \frac{\lambda \left( e^{t}-1\right) }{e^{\lambda t}-1}\frac{\log \left( 1+\lambda \left( e^{t}-1\right) \right) }{\lambda \left( e^{t}-1\right) }x^{k}\nonumber \\&=\sum _{k=0}^{n}S_{1}\left( n,k\right) \sum _{l=0}^{k}D_{l}\lambda ^{l}\sum _{m=l}^{k}\left( {\begin{array}{c}k\\ m\end{array}}\right) S_{2}\left( m,l\right) \frac{e^{t}-1}{t}\frac{\lambda t}{e^{\lambda t}-1}x^{k-m}, \end{aligned}$$

Now, we observe that

2.18 $$\begin{aligned} \frac{e^{t}-1}{t}\frac{\lambda t}{e^{\lambda t}-1}x^{k-m}&=\frac{e^{t}-1}{t}\sum _{j=0}^{k-m}B_{j}\lambda ^{j}\frac{t^{j}}{j!}x^{k-m}\nonumber \\&=\sum _{j=0}^{k-m}\left( {\begin{array}{c}k-m\\ j\end{array}}\right) B_{j}\lambda ^{j}\frac{e^{t}-1}{t}x^{k-m-j}\nonumber \\&=\sum _{j=0}^{k-m}\left( {\begin{array}{c}k-m\\ j\end{array}}\right) B_{j}\lambda ^{j}\sum _{i=0}^{k-m-j}\frac{1}{i+1}\left( {\begin{array}{c}k-m-j\\ i\end{array}}\right) x^{k-m-j-i}, \end{aligned}$$

where $B_{n}$ is the *n*-th Bernoulli number given by the generating function

$$\begin{aligned} \frac{t}{e^{t}-1}=\sum _{n=0}^{\infty }B_{n}\frac{t^{n}}{n!},\quad \left( \text {see Bayad and kim 2010; Kim et al. 2012, 2015}\right) . \end{aligned}$$

Thus, by (2.17) and (2.18), we get

2.19 $$\begin{aligned}&K_{n,4}\left( x\mid \lambda \right) \nonumber \\&=\sum _{k=0}^{n}S_{1}\left( n,k\right) \sum _{l=0}^{k}D_{l}\lambda ^{l}\sum _{m=0}^{k-l}\left( {\begin{array}{c}k\\ m\end{array}}\right) S_{2}\left( k-m,l\right) \nonumber \\&\quad \times \sum _{j=0}^{m}\left( {\begin{array}{c}m\\ j\end{array}}\right) B_{m-j}\lambda ^{m-j}\sum _{i=0}^{j}\frac{1}{j-i+1}\left( {\begin{array}{c}j\\ i\end{array}}\right) x^{i}\nonumber \\&=\sum _{i=0}^{n}\left( \sum _{k=i}^{n}\sum _{l=0}^{k-i}\sum _{m=i}^{k-l}\sum _{j=i}^{m}\frac{1}{j-i+1}\left( {\begin{array}{c}k\\ m\end{array}}\right) \right. \nonumber \\&\quad \times \left. \left( {\begin{array}{c}m\\ j\end{array}}\right) \left( {\begin{array}{c}j\\ i\end{array}}\right) \lambda ^{l+m-j}S_{1}\left( n,k\right) S_{2}\left( k-m,l\right) D_{l}B_{m-j}\right) x^{i}\nonumber \\&=\sum _{i=0}^{n}\left( \sum _{k=i}^{n}\sum _{l=0}^{k-i}\sum _{m=i}^{k-l}\sum _{j=i}^{m}\frac{1}{k+1}\left( {\begin{array}{c}k+1\\ m+1\end{array}}\right) \left( {\begin{array}{c}m+1\\ j+1\end{array}}\right) \left( {\begin{array}{c}j+1\\ i\end{array}}\right) \lambda ^{l+m-j}\right. \nonumber \\&\quad \times \left. S_{1}\left( n,k\right) S_{2}\left( k-m,l\right) D_{l}B_{m-j}\right) x^{i}. \end{aligned}$$

Therefore, by (2.19), we obtain the following theorem expressing $K_{n,4}\left( x\mid \lambda \right)$ in terms of the Stirling numbers of the first kind and of the second kind, Daehee numbers and Bernoulli numbers.

### **Theorem 4**

*For *$n\ge 0$*, we have*

$$\begin{aligned}&K_{n,4}\left( x\mid \lambda \right) \\&=\sum _{i=0}^{n}\left( \sum _{k=i}^{n}\sum _{l=0}^{k-i}\sum _{m=i}^{k-l}\sum _{j=i}^{m}\frac{1}{k+1}\left( {\begin{array}{c}k+1\\ m+1\end{array}}\right) \left( {\begin{array}{c}m+1\\ j+1\end{array}}\right) \left( {\begin{array}{c}j+1\\ i\end{array}}\right) \right. \\&\quad \times \left. \lambda ^{l+m-j}S_{1}\left( n,k\right) S_{2}\left( k-m,l\right) D_{l}B_{m-j}\right) x^{i}. \end{aligned}$$

From (2.8), we have

2.20 $$\begin{aligned} K_{n,3}\left( y\mid \lambda \right)&=\sum _{l=0}^{n}\left( {\begin{array}{c}n\\ l\end{array}}\right) \left( y\right) _{l}\left\langle \left. \frac{\log \left( 1+\lambda t\right) }{\lambda \log \left( 1+t\right) }\right| x^{n-l}\right\rangle \nonumber \\&=\sum _{l=0}^{n}\left( {\begin{array}{c}n\\ l\end{array}}\right) \left( y\right) _{l}\left\langle \left. \sum _{i=0}^{\infty }K_{i,3}\left( \lambda \right) \frac{t^{i}}{i!}\right| x^{n-l}\right\rangle \nonumber \\&=\sum _{l=0}^{n}\left( {\begin{array}{c}n\\ l\end{array}}\right) \left( y\right) _{l}K_{n-l,3}\left( \lambda \right) . \end{aligned}$$

Thus, by (2.20), we get

2.21 $$\begin{aligned} K_{n,3}\left( x\mid \lambda \right) =\sum _{l=0}^{n}\left( {\begin{array}{c}n\\ l\end{array}}\right) K_{n-l,3}\left( \lambda \right) \left( x\right) _{l}. \end{aligned}$$

From (2.10), we have

2.22 $$\begin{aligned} K_{n,4}\left( y\mid \lambda \right)&=\sum _{l=0}^{n}\left( {\begin{array}{c}n\\ l\end{array}}\right) \left( y\right) _{l}\left\langle \left. \frac{\log \left( 1+\lambda t\right) }{\left( 1+t\right) ^{\lambda }-1}\right| x^{n-l}\right\rangle \nonumber \\&=\sum _{l=0}^{n}\left( {\begin{array}{c}n\\ l\end{array}}\right) \left( y\right) _{l}\left\langle \left. \sum _{i=0}^{\infty }K_{i,4}\left( \lambda \right) \frac{t^{i}}{i!}\right| x^{n-l}\right\rangle \nonumber \\&=\sum _{l=0}^{n}\left( {\begin{array}{c}n\\ l\end{array}}\right) \left( y\right) _{l}K_{n-l,4}\left( \lambda \right) . \end{aligned}$$

By (2.22), we get

2.23 $$\begin{aligned} K_{n,4}\left( x\mid \lambda \right) =\sum _{l=0}^{n}\left( {\begin{array}{c}n\\ l\end{array}}\right) K_{n-l,4}\left( \lambda \right) \left( x\right) _{l},\quad \left( n\ge 0\right) . \end{aligned}$$

From (2.19), we have

2.24 $$\begin{aligned} K_{n,3}\left( y\mid \lambda \right)&=\left\langle \left. \frac{\log \left( 1+\lambda t\right) }{\lambda \log \left( 1+t\right) }\left( 1+t\right) ^{y}\right| x^{n}\right\rangle \nonumber \\&=\left\langle \left. \frac{\log \left( 1+\lambda t\right) }{\lambda t}\right| \frac{t}{\log \left( 1+t\right) }\left( 1+t\right) ^{y}x^{n}\right\rangle \nonumber \\&=\sum _{l=0}^{n}\left( {\begin{array}{c}n\\ l\end{array}}\right) b_{l}\left( y\right) \left\langle \left. \frac{\log \left( 1+\lambda t\right) }{\lambda t}\right| x^{n-l}\right\rangle \nonumber \\&=\sum _{l=0}^{n}\left( {\begin{array}{c}n\\ l\end{array}}\right) b_{l}\left( y\right) \left\langle \left. \sum _{m=0}^{\infty }D_{m}\lambda ^{m}\frac{t^{m}}{m!}\right| x^{n-l}\right\rangle \nonumber \\&=\sum _{l=0}^{n}\left( {\begin{array}{c}n\\ l\end{array}}\right) b_{l}\left( y\right) D_{n-l}\lambda ^{n-l}. \end{aligned}$$

Thus, by (2.24), we get

2.25 $$\begin{aligned} K_{n,3}\left( x\mid \lambda \right) =\sum _{l=0}^{n}\left( {\begin{array}{c}n\\ l\end{array}}\right) D_{n-l}\lambda ^{n-l}b_{l}\left( x\right) ,\quad \left( n\ge 0\right) . \end{aligned}$$

From (2.10), we can also derive the following equation:

2.26 $$\begin{aligned} K_{n,4}\left( x\mid \lambda \right)&=\left\langle \left. \frac{\log \left( 1+\lambda t\right) }{\left( 1+t\right) ^{\lambda }-1}\left( 1+t\right) ^{y}\right| x^{n}\right\rangle \nonumber \\&=\left\langle \left. \frac{\log \left( 1+\lambda t\right) }{\lambda t}\right| \frac{\lambda t}{\left( 1+t\right) ^{\lambda }-1}\left( 1+t\right) ^{y}x^{n}\right\rangle \nonumber \\&=\left\langle \left. \frac{\log \left( 1+\lambda t\right) }{\lambda t}\right| \sum _{l=0}^{\infty }K_{l}\left( y\mid \lambda \right) \frac{t^{l}}{l!}x^{n}\right\rangle \nonumber \\&=\sum _{l=0}^{n}\left( {\begin{array}{c}n\\ l\end{array}}\right) K_{l}\left( y\mid \lambda \right) \left\langle \left. \frac{\log \left( 1+\lambda t\right) }{\lambda t}\right| x^{n-l}\right\rangle \nonumber \\&=\sum _{l=0}^{n}\left( {\begin{array}{c}n\\ l\end{array}}\right) K_{l}\left( y\mid \lambda \right) \left\langle \left. \sum _{m=0}^{\infty }D_{m}\lambda ^{m}\frac{t^{m}}{m!}\right| x^{n-l}\right\rangle \nonumber \\&=\sum _{l=0}^{n}\left( {\begin{array}{c}n\\ l\end{array}}\right) K_{l}\left( y\mid \lambda \right) D_{n-l}\lambda ^{n-l}. \end{aligned}$$

Thus, by (2.26), we get

2.27 $$\begin{aligned} K_{n,4}\left( x\mid \lambda \right) =\sum _{l=0}^{n}\left( {\begin{array}{c}n\\ l\end{array}}\right) D_{n-l}\lambda ^{n-l}K_{l}\left( x\mid \lambda \right) . \end{aligned}$$

Therefore, by (2.21), (2.23), (2.25) and (2.27), we obtain the following theorem expressing $K_{n,3}\left( x\mid \lambda \right)$ and $K_{n,4}\left( x\mid \lambda \right)$ both in terms of falling factorial polynomials. Also, we express $K_{n,3}\left( x\mid \lambda \right)$ and $K_{n,4}\left( x\mid \lambda \right)$ respectively by Bernoulli polynomials of the second kind and Korobov polynomials of the first kind.

### **Theorem 5**

*For*$n\ge 0$*, we have*

$$\begin{aligned} K_{n,3}\left( x\mid \lambda \right) =\sum _{l=0}^{n}\left( {\begin{array}{c}n\\ l\end{array}}\right) K_{n-l,3}\left( \lambda \right) \left( x\right) _{l}=\sum _{l=0}^{n}\left( {\begin{array}{c}n\\ l\end{array}}\right) D_{n-l}\lambda ^{n-l}b_{l}\left( x\right) , \end{aligned}$$

and

$$\begin{aligned} K_{n,4}\left( x\mid \lambda \right) =\sum _{l=0}^{n}\left( {\begin{array}{c}n\\ l\end{array}}\right) K_{n-l,4}\left( \lambda \right) \left( x\right) _{l}=\sum _{l=0}^{n}\left( {\begin{array}{c}n\\ l\end{array}}\right) D_{n-l}\lambda ^{n-l}K_{l}\left( x\mid \lambda \right) . \end{aligned}$$

It is easy to see that

2.28 $$\begin{aligned} x^{n}\sim \left( 1,t\right) ,\quad \frac{\lambda t}{\log \left( 1+\lambda \left( e^{t}-1\right) \right) }K_{n,3}\left( x\mid \lambda \right) \sim \left( 1,e^{t}-1\right) . \end{aligned}$$

For $n\ge 1$, we have

2.29 $$\begin{aligned} \frac{\lambda t}{\log \left( 1+\lambda \left( e^{t}-1\right) \right) }K_{n,3}\left( x\mid \lambda \right)&=x\left( \frac{t}{e^{t}-1}\right) ^{n}x^{-1}x^{n}\nonumber \\&=xB_{n-1}^{\left( n\right) }\left( x\right) \nonumber \\&=\sum _{k=0}^{n-1}\left( {\begin{array}{c}n-1\\ k\end{array}}\right) B_{k}^{\left( n\right) }x^{n-k}\nonumber \\&=\sum _{k=1}^{n}\left( {\begin{array}{c}n-1\\ k-1\end{array}}\right) B_{n-k}^{\left( n\right) }x^{k}. \end{aligned}$$

Thus, by (2.29), we get

2.30 $$\begin{aligned}&K_{n,3}\left( x\mid \lambda \right) \nonumber \\&=\sum _{k=1}^{n}\left( {\begin{array}{c}n-1\\ k-1\end{array}}\right) B_{n-k}^{\left( n\right) }\frac{\log \left( 1+\lambda \left( e^{t}-1\right) \right) }{\lambda t}x^{k}\nonumber \\&=\sum _{k=1}^{n}\left( {\begin{array}{c}n-1\\ k-1\end{array}}\right) B_{n-k}^{\left( n\right) }\sum _{l=0}^{k}\sum _{m=0}^{k-l}\sum _{j=0}^{m}\frac{1}{k+1}\left( {\begin{array}{c}k+1\\ m+1\end{array}}\right) \left( {\begin{array}{c}m+1\\ j\end{array}}\right) S_{2}\left( k-m,l\right) D_{l}\lambda ^{l}x^{j}\nonumber \\&=\sum _{k=0}^{n}\sum _{l=0}^{k}\sum _{m=0}^{k-l}\sum _{j=0}^{m}\frac{1}{k+1}\left( {\begin{array}{c}n-1\\ k-1\end{array}}\right) \left( {\begin{array}{c}k+1\\ m+1\end{array}}\right) \left( {\begin{array}{c}m+1\\ j\end{array}}\right) S_{2}\left( k-m,l\right) \lambda ^{l}D_{l}B_{n-k}^{\left( n\right) }x^{j}\nonumber \\&=\sum _{j=0}^{n}\left( \sum _{k=j}^{n}\sum _{l=0}^{k-j}\sum _{m=j}^{k-l}\frac{1}{k+1}\left( {\begin{array}{c}n-1\\ k-1\end{array}}\right) \left( {\begin{array}{c}k+1\\ m+1\end{array}}\right) \left( {\begin{array}{c}m+1\\ j\end{array}}\right) S_{2}\left( k-m,l\right) \lambda ^{l}D_{l}B_{n-k}^{\left( n\right) }\right) x^{j}. \end{aligned}$$

From (2.3), we note that

2.31 $$\begin{aligned} x^{n}\sim \left( 1,t\right) ,\quad \frac{e^{\lambda t}-1}{\log \left( 1+\lambda \left( e^{t}-1\right) \right) }K_{n,4}\left( x\mid \lambda \right) \sim \left( 1,e^{t}-1\right) . \end{aligned}$$

For $n\ge 1$, by (2.31), we get

2.32 $$\begin{aligned} \frac{e^{\lambda t}-1}{\log \left( 1+\lambda \left( e^{t}-1\right) \right) }K_{n,4}\left( x\mid \lambda \right)&=x\left( \frac{t}{e^{t}-1}\right) ^{n}x^{-1}x^{n}=xB_{n-1}^{\left( n\right) }\left( x\right) \nonumber \\&=\sum _{k=1}^{n}\left( {\begin{array}{c}n-1\\ k-1\end{array}}\right) B_{n-k}^{\left( n\right) }x^{k}. \end{aligned}$$

Thus, by (2.32), we have

2.33 $$\begin{aligned}&K_{n,4}\left( x\mid \lambda \right) \nonumber \\&=\sum _{k=1}^{n}\left( {\begin{array}{c}n-1\\ k-1\end{array}}\right) B_{n-k}^{\left( n\right) }\frac{\log \left( 1+\lambda \left( e^{t}-1\right) \right) }{e^{\lambda t}-1}x^{k}\nonumber \\&=\sum _{k=1}^{n}\left( {\begin{array}{c}n-1\\ k-1\end{array}}\right) B_{n-k}^{\left( n\right) }\sum _{l=0}^{k}\sum _{m=0}^{k-l}\sum _{j=0}^{m}\sum _{i=0}^{j}\frac{1}{j-i+1}\left( {\begin{array}{c}k\\ m\end{array}}\right) \nonumber \\&\quad \times \left( {\begin{array}{c}m\\ j\end{array}}\right) \left( {\begin{array}{c}j\\ i\end{array}}\right) \lambda ^{l+m-j}S_{2}\left( k-m,l\right) D_{l}B_{m-j}x^{i}\nonumber \\&=\sum _{i=0}^{n}\left( \sum _{k=i}^{n}\sum _{l=0}^{k-i}\sum _{m=i}^{k-l}\sum _{j=i}^{m}\frac{1}{j-i+1}\left( {\begin{array}{c}n-1\\ k-1\end{array}}\right) \left( {\begin{array}{c}k\\ m\end{array}}\right) \left( {\begin{array}{c}m\\ j\end{array}}\right) \left( {\begin{array}{c}j\\ i\end{array}}\right) \right. \nonumber \\&\quad \times \left. \lambda ^{l+m-j}S_{2}\left( k-m,l\right) D_{l}B_{m-j}B_{n-k}^{\left( n\right) }\right) x^{i}\nonumber \\&=\sum _{i=0}^{n}\left( \sum _{k=i}^{n}\sum _{l=0}^{k-i}\sum _{m=i}^{k-l}\sum _{j=i}^{m}\frac{1}{k+1}\left( {\begin{array}{c}n-1\\ k-1\end{array}}\right) \left( {\begin{array}{c}k+1\\ m+1\end{array}}\right) \left( {\begin{array}{c}m+1\\ j+1\end{array}}\right) \right. \nonumber \\&\quad \times \left. \left( {\begin{array}{c}j+1\\ i\end{array}}\right) \lambda ^{l+m-j}S_{2}\left( k-m,l\right) D_{l}B_{m-j}B_{n-k}^{\left( n\right) }\right) x^{i}. \end{aligned}$$

Therefore, by (2.30) and (2.33), we obtain the following theorem.

### **Theorem 6**

*For*$n\ge 0$*, we have*

$$\begin{aligned}&K_{n,3}\left( x\mid \lambda \right) \\&=\sum _{j=0}^{n}\left( \sum _{k=j}^{n}\sum _{l=0}^{k-j}\sum _{m=j}^{k-l}\frac{1}{k+1}\left( {\begin{array}{c}n-1\\ k-1\end{array}}\right) \left( {\begin{array}{c}k+1\\ m+1\end{array}}\right) \left( {\begin{array}{c}m+1\\ j\end{array}}\right) S_{2}\left( k-m,l\right) \lambda ^{l}D_{l}B_{n-k}^{\left( n\right) }\right) x^{j} \end{aligned}$$

and

$$\begin{aligned}&K_{n,4}\left( x\mid \lambda \right) \\&=\sum _{i=0}^{n}\left( \sum _{k=i}^{n}\sum _{l=0}^{k-i}\sum _{m=i}^{k-l}\sum _{j=i}^{m}\frac{1}{k+1}\left( {\begin{array}{c}n-1\\ k-1\end{array}}\right) \left( {\begin{array}{c}k+1\\ m+1\end{array}}\right) \left( {\begin{array}{c}m+1\\ j+1\end{array}}\right) \right. \\&\quad \times \left. \left( {\begin{array}{c}j+1\\ i\end{array}}\right) \lambda ^{l+m-j}S_{2}\left( k-m,l\right) D_{l}B_{m-j}B_{n-k}^{\left( n\right) }\right) x^{i} \end{aligned}$$

For $s_{n}\left( x\right) \sim \left( g\left( t\right) ,f\left( t\right) \right) ,$ we note that Sheffer identity is given by

2.34 $$\begin{aligned} s_{n}\left( x+y\right) =\sum _{j=0}^{n}\left( {\begin{array}{c}n\\ j\end{array}}\right) s_{j}\left( x\right) p_{n-j}\left( y\right) ,\quad \text {where }p_{n}\left( x\right) =g\left( t\right) s_{n}\left( x\right) . \end{aligned}$$

By (2.2) and (2.34), we get

2.35 $$\begin{aligned} K_{n,3}\left( x+y\mid \lambda \right) =\sum _{j=0}^{n}\left( {\begin{array}{c}n\\ j\end{array}}\right) K_{j,3}\left( x\mid \lambda \right) \left( y\right) _{n-j}, \end{aligned}$$

where $$p_{n}\left( x\right) =\frac{\lambda t}{\log \left( 1+\lambda \left( e^{t}-1\right) \right) }K_{n,3}\left( x\mid \lambda \right) =\left( x\right) _{n},\quad \left( n\ge 0\right)$$.

From (2.3) and (2.34), we have

2.36 $$\begin{aligned} K_{n,4}\left( x+y\mid \lambda \right) =\sum _{j=0}^{n}\left( {\begin{array}{c}n\\ j\end{array}}\right) K_{j,4}\left( x\mid \lambda \right) \left( y\right) _{n-j}, \end{aligned}$$

where $$p_{n}\left( x\right) =\frac{e^{\lambda t}-1}{\log \left( 1+\lambda \left( e^{t}-1\right) \right) }K_{n,4}\left( x\mid \lambda \right) =\left( x\right) _{n}$$.

By (1.10), we see that

2.37 $$\begin{aligned} \left( e^{t}-1\right) K_{n,3}\left( x\mid \lambda \right) =nK_{n-1,3}\left( x\mid \lambda \right) , \end{aligned}$$

and

2.38 $$\begin{aligned} \left( e^{t}-1\right) K_{n,3}\left( x\mid \lambda \right)&=e^{t}K_{n,3}\left( x\mid \lambda \right) -K_{n,3}\left( x\mid \lambda \right) \nonumber \\&=K_{n,3}\left( x+1\mid \lambda \right) -K_{n,3}\left( x\mid \lambda \right) . \end{aligned}$$

From (2.37) and (2.38), we have

2.39 $$\begin{aligned} nK_{n-1,3}\left( x\mid \lambda \right) =K_{n,3}\left( x+1\mid \lambda \right) -K_{n,3}\left( x\mid \lambda \right) . \end{aligned}$$

By (1.10) and (2.3), we get

2.40 $$\begin{aligned} \left( e^{t}-1\right) K_{n,4}\left( x\mid \lambda \right) =nK_{n-1,4}\left( x\mid \lambda \right) . \end{aligned}$$

Thus, by (2.40), we have

2.41 $$\begin{aligned} K_{n,4}\left( x+1\mid \lambda \right) -K_{n,4}\left( x\mid \lambda \right) =nK_{n-1,4}\left( x\mid \lambda \right) . \end{aligned}$$

Therefore, by (2.35), (2.36), (2.39) and (2.41), we obtain the following theorem.

### **Theorem 7**

*For*$n\ge 0$*, we have*

$$\begin{aligned} K_{n,3}\left( x+y\mid \lambda \right)&=\sum _{j=0}^{n}\left( {\begin{array}{c}n\\ j\end{array}}\right) K_{j,3}\left( x\mid \lambda \right) \left( y\right) _{n-j},\\ K_{n,4}\left( x+y\mid \lambda \right)&=\sum _{j=0}^{n}\left( {\begin{array}{c}n\\ j\end{array}}\right) K_{j,4}\left( x\mid \lambda \right) \left( y\right) _{n-j},\\ nK_{n-1,3}\left( x\mid \lambda \right)&=K_{n,3}\left( x+1\mid \lambda \right) -K_{n,3}\left( x\mid \lambda \right) , \end{aligned}$$

and

$$\begin{aligned} nK_{n-1,4}\left( x\mid \lambda \right) =K_{n,4}\left( x+1\mid \lambda \right) -K_{n,4}\left( x\mid \lambda \right) . \end{aligned}$$

For $s_{n}\left( x\right) \sim \left( g\left( t\right) ,f\left( t\right) \right)$, we note that

2.42 $$\begin{aligned} \frac{d}{dx}s_{n}\left( x\right) =\sum _{l=0}^{n-1}\left( {\begin{array}{c}n\\ l\end{array}}\right) \left\langle \left. \overline{f}\left( t\right) \right| x^{n-l}\right\rangle s_{l}\left( x\right) . \end{aligned}$$

For $K_{n,3}\left( x\mid \lambda \right) \sim \left( \frac{\lambda t}{\log \left( 1+\lambda \left( e^{t}-1\right) \right) },e^{t}-1\right)$, by (2.42), we get

2.43 $$\begin{aligned} \left\langle \left. \overline{f}\left( t\right) \right| x^{n-l}\right\rangle&=\left\langle \left. \log \left( 1+t\right) \right| x^{n-l}\right\rangle \nonumber \\&=\left\langle \left. \sum _{m=1}^{\infty }\left( -1\right) ^{m-1}\left( m-1\right) !\frac{t^{m}}{m!}\right| x^{n-l}\right\rangle \nonumber \\&=\left( -1\right) ^{n-l-1}\left( n-l-1\right) !. \end{aligned}$$

Thus, by (2.42) and (2.43), we have

2.44 $$\begin{aligned} \frac{d}{dx}K_{n,3}\left( x\mid \lambda \right)&=\sum _{l=0}^{n-1}\left( {\begin{array}{c}n\\ l\end{array}}\right) \left( -1\right) ^{n-l-1}\left( n-l-1\right) !K_{l,3}\left( x\mid \lambda \right) \nonumber \\&=n!\sum _{l=0}^{n-1}\frac{\left( -1\right) ^{n-l-1}}{l!\left( n-l\right) }K_{l,3}\left( x\mid \lambda \right) . \end{aligned}$$

By the same method as (2.44), we get

2.45 $$\begin{aligned} \frac{d}{dx}K_{n,4}\left( x\mid \lambda \right) =n!\sum _{l=0}^{n-1}\frac{\left( -1\right) ^{n-l-1}}{l!\left( n-l\right) }K_{l,4}\left( x\mid \lambda \right) . \end{aligned}$$

Therefore, by (2.44) and (2.45), we obtain the following theorem.

### **Theorem 8**

*For*$n\ge 1$*, we have*

$$\begin{aligned} \frac{d}{dx}K_{n,3}\left( x\mid \lambda \right) =n!\sum _{l=0}^{n-1}\frac{\left( -1\right) ^{n-l-1}}{l!\left( n-l\right) }K_{l,3}\left( x\mid \lambda \right) , \end{aligned}$$

and

$$\begin{aligned} \frac{d}{dx}K_{n,4}\left( x\mid \lambda \right) =n!\sum _{l=0}^{n-1}\frac{\left( -1\right) ^{n-l-1}}{l!\left( n-l\right) }K_{l,4}\left( x\mid \lambda \right) . \end{aligned}$$

Let $n\ge 1$. Then, by (1.6), (2.1) and (2.3), we get

2.46 $$\begin{aligned}&K_{n,3}\left( y\mid \lambda \right) \nonumber \\&=\left\langle \left. \frac{\log \left( 1+\lambda t\right) }{\lambda \log \left( 1+t\right) }\left( 1+t\right) ^{y}\right| x^{n}\right\rangle \nonumber \\&=\left\langle \left. \partial _{t}\left( \frac{\log \left( 1+\lambda t\right) }{\lambda \log \left( 1+t\right) }\left( 1+t\right) ^{y}\right) \right| x^{n-1}\right\rangle \nonumber \\&=\left\langle \left. \frac{\log \left( 1+\lambda t\right) }{\lambda \log \left( 1+t\right) }\partial _{t}\left( 1+t\right) ^{y}\right| x^{n-1}\right\rangle +\left\langle \left. \left( \partial _{t}\frac{\log \left( 1+\lambda t\right) }{\log \left( 1+t\right) }\right) \left( 1+t\right) ^{y}\right| x^{n-1}\right\rangle . \end{aligned}$$

We observe that

2.47 $$\begin{aligned} \left\langle \left. \frac{\log \left( 1+\lambda t\right) }{\lambda \log \left( 1+t\right) }\partial _{t}\left( 1+t\right) ^{y}\right| x^{n-1}\right\rangle&=y\left\langle \left. \frac{\log \left( 1+\lambda t\right) }{\lambda \log \left( 1+t\right) }\left( 1+t\right) ^{y-1}\right| x^{n-1}\right\rangle \nonumber \\&=yK_{n-1,3}\left( y-1\mid \lambda \right) , \end{aligned}$$

and

2.48 $$\begin{aligned} \partial _{t}\left( \frac{\log \left( 1+\lambda t\right) }{\lambda \log \left( 1+t\right) }\right)&=\frac{\frac{\lambda }{1+\lambda t}\cdot \lambda \log \left( 1+t\right) -\log \left( 1+\lambda t\right) \frac{\lambda }{1+t}}{\left( \lambda \log \left( 1+t\right) \right) ^{2}}\nonumber \\&=\frac{t}{\log \left( 1+t\right) }\frac{1}{t}\left\{ \frac{1}{1+\lambda t}-\frac{\log \left( 1+\lambda t\right) }{\lambda \log \left( 1+t\right) }\left( 1+t\right) ^{-1}\right\} . \end{aligned}$$

By (2.48), we get

2.49 $$\begin{aligned}&\left\langle \left. \left( \partial _{t}\left( \frac{\log \left( 1+\lambda t\right) }{\log \left( 1+t\right) }\right) \right) \left( 1+t\right) ^{y}\right| x^{n-1}\right\rangle \nonumber \\&=\left\langle \left. \frac{t}{\log \left( 1+t\right) }\frac{1}{t}\left\{ \frac{1}{1+\lambda t}-\frac{\log \left( 1+\lambda t\right) }{\lambda \log \left( 1+t\right) }\left( 1+t\right) ^{-1}\right\} \left( 1+t\right) ^{y}\right| x^{n-1}\right\rangle \nonumber \\&=\frac{1}{n}\left\langle \left. \left\{ \frac{1}{1+\lambda t}-\frac{\log \left( 1+\lambda t\right) }{\lambda \log \left( 1+t\right) }\left( 1+t\right) ^{-1}\right\} \left( 1+t\right) ^{y}\right| \frac{t}{\log \left( 1+t\right) }x^{n}\right\rangle \nonumber \\&=\frac{1}{n}\left\langle \left. \left\{ \frac{1}{1+\lambda t}-\frac{\log \left( 1+\lambda t\right) }{\lambda \log \left( 1+t\right) }\left( 1+t\right) ^{-1}\right\} \left( 1+t\right) ^{y}\right| \sum _{l=0}^{\infty }b_{l}\frac{t^{l}}{l!}x^{n}\right\rangle \nonumber \\&=\frac{1}{n}\sum _{l=0}^{n}\left( {\begin{array}{c}n\\ l\end{array}}\right) b_{l}\left\{ \left\langle \left. \frac{1}{1+\lambda t}\left( 1+t\right) ^{y}\right| x^{n-l}\right\rangle -\left\langle \left. \frac{\log \left( 1+\lambda t\right) }{\lambda \log \left( 1+t\right) }\left( 1+t\right) ^{y-1}\right| x^{n-l}\right\rangle \right\} \nonumber \\&=\frac{1}{n}\sum _{l=0}^{n}\left( {\begin{array}{c}n\\ l\end{array}}\right) b_{l}\left\{ \left\langle \left( 1+t\right) ^{y}\left| \sum _{m=0}^{\infty }\left( -\lambda t\right) ^{m}x^{n-l}\right. \right\rangle -K_{n-l,3}\left( y-1\mid \lambda \right) \right\} \nonumber \\&=\frac{1}{n}\sum _{l=0}^{n}\left( {\begin{array}{c}n\\ l\end{array}}\right) b_{l}\left\{ \sum _{m=0}^{n-l}\left( -\lambda \right) ^{m}\left( n-l\right) _{m}\left\langle \left. \left( 1+t\right) ^{y}\right| x^{n-l-m}\right\rangle -K_{n-l,3}\left( y-1\mid \lambda \right) \right\} \nonumber \\&=\frac{1}{n}\sum _{l=0}^{n}\left( {\begin{array}{c}n\\ l\end{array}}\right) b_{l}\left\{ \sum _{m=0}^{n-l}\left( -\lambda \right) ^{m}\left( n-l\right) _{m}\left( y\right) _{n-l-m}-K_{n-l,3}\left( y-1\mid \lambda \right) \right\} . \end{aligned}$$

For $n\ge 1$, from (2.46), (2.47) and (2.49), we have

2.50 $$\begin{aligned}&K_{n,3}\left( x\mid \lambda \right) -xK_{n-1,3}\left( x-1\mid \lambda \right) \nonumber \\&=\frac{1}{n}\sum _{l=0}^{n}\left( {\begin{array}{c}n\\ l\end{array}}\right) b_{l}\left\{ \sum _{m=0}^{n-l}\left( -\lambda \right) ^{m}\left( n-l\right) _{m}\left( x\right) _{n-l-m}-K_{n-l,3}\left( x-1\mid \lambda \right) \right\} . \end{aligned}$$

Therefore, by (2.50), we obtain the following theorem.

### **Theorem 9**

*For*$n\ge 1$*, we have*

$$\begin{aligned}&K_{n,3}\left( x\mid \lambda \right) -xK_{n-1,3}\left( x-1\mid \lambda \right) \\&=\frac{1}{n}\sum _{l=0}^{n}\left( {\begin{array}{c}n\\ l\end{array}}\right) b_{l}\left\{ \sum _{m=0}^{n-l}\left( -\lambda \right) ^{m}\left( n-l\right) _{m}\left( x\right) _{n-l-m}-K_{n-l,3}\left( x-1\mid \lambda \right) \right\} . \end{aligned}$$

### *Remark*

We note that

$$\begin{aligned} b_{n}\left( x\right) -xb_{n-1}\left( x-1\right)&=\lim _{\lambda \rightarrow 0}\left( K_{n,3}\left( x\mid \lambda \right) -xK_{n-1,3}\left( x-1\mid \lambda \right) \right) \\&=\frac{1}{n}\sum _{l=0}^{n}\left( {\begin{array}{c}n\\ l\end{array}}\right) b_{l}\left( \left( x\right) _{n-l}-b_{n-l}\left( x-1\right) \right) . \end{aligned}$$

From (1.6) and (2.2), we have

2.51 $$\begin{aligned} K_{n,4}\left( y\mid \lambda \right)&=\left\langle \left. \frac{\log \left( 1+\lambda t\right) }{\left( 1+t\right) ^{\lambda }-1}\left( 1+t\right) ^{y}\right| x^{n}\right\rangle \nonumber \\&=\left\langle \left. \partial _{t}\left( \frac{\log \left( 1+\lambda t\right) }{\left( 1+t\right) ^{\lambda }-1}\left( 1+t\right) ^{y}\right) \right| x^{n-1}\right\rangle \nonumber \\&=\left\langle \left. \frac{\log \left( 1+\lambda t\right) }{\left( 1+t\right) ^{\lambda }-1}\partial _{t}\left( 1+t\right) ^{y}\right| x^{n-1}\right\rangle \nonumber \\&\quad +\left\langle \left. \left( \partial _{t}\left( \frac{\log \left( 1+\lambda t\right) }{\left( 1+t\right) ^{\lambda }-1}\right) \right) \left( 1+t\right) ^{y}\right| x^{n-1}\right\rangle , \end{aligned}$$

where $$n\ge 1$$.

We note that

2.52 $$\begin{aligned}&\left\langle \left. \frac{\log \left( 1+\lambda t\right) }{\left( 1+t\right) ^{\lambda }-1}\left( \partial _{t}\left( 1+t\right) ^{y}\right) \right| x^{n-1}\right\rangle \nonumber \\&=y\left\langle \left. \frac{\log \left( 1+\lambda t\right) }{\left( 1+t\right) ^{\lambda }-1}\left( 1+t\right) ^{y-1}\right| x^{n-1}\right\rangle \nonumber \\&=yK_{n-1,4}\left( y-1\mid \lambda \right) . \end{aligned}$$

Now, we observe that

2.53 $$\begin{aligned}&\partial _{t}\left( \frac{\log \left( 1+\lambda t\right) }{\left( 1+t\right) ^{\lambda }-1}\right) \nonumber \\&=\frac{\frac{\lambda }{1+\lambda t}\left( \left( 1+t\right) ^{\lambda }-1\right) -\log \left( 1+\lambda t\right) \lambda \left( 1+t\right) ^{\lambda -1}}{\left( \left( 1+t\right) ^{\lambda }-1\right) ^{2}}\nonumber \\&=\frac{\lambda t}{\left( 1+t\right) ^{\lambda }-1}\frac{1}{t}\left\{ \frac{1}{1+\lambda t}-\frac{\log \left( 1+\lambda t\right) }{\left( 1+t\right) ^{\lambda }-1}\left( 1+t\right) ^{\lambda -1}\right\} . \end{aligned}$$

Thus, by (2.53), we get

2.54 $$\begin{aligned}&\left\langle \left. \left( \partial _{t}\frac{\log \left( 1+\lambda t\right) }{\left( 1+t\right) ^{\lambda }-1}\right) \left( 1+t\right) ^{y}\right| x^{n-1}\right\rangle \nonumber \\&=\left\langle \left. \frac{\lambda t}{\left( 1+t\right) ^{\lambda }-1}\frac{1}{t}\left\{ \frac{1}{1+\lambda t}-\frac{\log \left( 1+\lambda t\right) }{\left( 1+t\right) ^{\lambda }-1}\left( 1+t\right) ^{\lambda -1}\right\} \left( 1+t\right) ^{y}\right| x^{n-1}\right\rangle \nonumber \\&=\frac{1}{n}\left\langle \left. \frac{\lambda t}{\left( 1+t\right) ^{\lambda }-1}\left\{ \frac{1}{1+\lambda t}-\frac{\log \left( 1+\lambda t\right) }{\left( 1+t\right) ^{\lambda }-1}\left( 1+t\right) ^{\lambda -1}\right\} \left( 1+t\right) ^{y}\right| x^{n}\right\rangle \nonumber \\&=\frac{1}{n}\left\langle \left. \left\{ \frac{1}{1+\lambda t}-\frac{\log \left( 1+\lambda t\right) }{\left( 1+t\right) ^{\lambda }-1}\left( 1+t\right) ^{\lambda -1}\right\} \left( 1+t\right) ^{y}\right| \frac{\lambda t}{\left( 1+t\right) ^{\lambda }-1}x^{n}\right\rangle \nonumber \\&=\frac{1}{n}\left\langle \left. \left\{ \frac{1}{1+\lambda t}-\frac{\log \left( 1+\lambda t\right) }{\left( 1+t\right) ^{\lambda }-1}\left( 1+t\right) ^{\lambda -1}\right\} \left( 1+t\right) ^{y}\right| \sum _{l=0}^{\infty }K_{l}\left( \lambda \right) \frac{t^{l}}{l!}x^{n}\right\rangle \nonumber \\&=\frac{1}{n}\sum _{l=0}^{n}\left( {\begin{array}{c}n\\ l\end{array}}\right) K_{l}\left( \lambda \right) \left\langle \left. \frac{1}{1+\lambda t}\left( 1+t\right) ^{y}\right| x^{n-l}\right\rangle \nonumber \\&\quad -\left\langle \left. \frac{\log \left( 1+\lambda t\right) }{\left( 1+t\right) ^{\lambda }-1}\left( 1+t\right) ^{y+\lambda -1}\right| x^{n-l}\right\rangle \nonumber \\&=\frac{1}{n}\sum _{l=0}^{n}\left( {\begin{array}{c}n\\ l\end{array}}\right) K_{l}\left( \lambda \right) \left\{ \left\langle \left. \left( 1+t\right) ^{y}\right| \sum _{m=0}^{\infty }\left( -\lambda t\right) ^{m}x^{n-l}\right\rangle -K_{n-1,4}\left( y+\lambda -1\mid \lambda \right) \right\} \nonumber \\&=\frac{1}{n}\sum _{l=0}^{n}\left( {\begin{array}{c}n\\ l\end{array}}\right) K_{l}\left( \lambda \right) \left\{ \sum _{m=0}^{n-l}\left( -\lambda \right) ^{m}\left( n-l\right) _{m}\left\langle \left. \left( 1+t\right) ^{y}\right| x^{n-l-m}\right\rangle \right. \nonumber \\&\quad \left. -K_{n-1,4}\left( y+\lambda -1\mid \lambda \right) \right\} \nonumber \\&=\frac{1}{n}\sum _{l=0}^{n}\left( {\begin{array}{c}n\\ l\end{array}}\right) K_{l}\left( \lambda \right) \left\{ \sum _{m=0}^{n-l}\left( -\lambda \right) ^{m}\left( n-l\right) _{m}\left( y\right) _{n-l-m}-K_{n-l,4}\left( y+\lambda -1\mid \lambda \right) \right\} \nonumber \\&=\frac{1}{n}\sum _{l=0}^{n}\left( {\begin{array}{c}n\\ l\end{array}}\right) K_{l}\left( \lambda \right) \left\{ \sum _{m=0}^{n-l}\left( -\lambda \right) ^{m}\left( n-l\right) _{m}\left( y\right) _{n-l-m}-K_{n-l,4}\left( y+\lambda -1\mid \lambda \right) \right\} . \end{aligned}$$

From (2.51), (2.52) and (2.54), we have

2.55 $$\begin{aligned}&K_{n,4}\left( x\mid \lambda \right) -xK_{n-1,4}\left( x-1\mid \lambda \right) \nonumber \\&=\frac{1}{n}\sum _{l=0}^{n}\left( {\begin{array}{c}n\\ l\end{array}}\right) K_{l}\left( \lambda \right) \left\{ \sum _{m=0}^{n-l}\left( -\lambda \right) ^{m}\left( n-l\right) _{m}\left( x\right) _{n-l-m}-K_{n-l,4}\left( x+\lambda -1\mid \lambda \right) \right\} . \end{aligned}$$

Therefore, by (2.55), we obtain the following theorem.

### **Theorem 10**

*For*$n\ge 1$*, we have*

$$\begin{aligned}&K_{n,4}\left( x\mid \lambda \right) -xK_{n-1,4}\left( x-1\mid \lambda \right) \\&=\frac{1}{n}\sum _{l=0}^{n}\left( {\begin{array}{c}n\\ l\end{array}}\right) K_{l}\left( \lambda \right) \left\{ \sum _{m=0}^{n-l}\left( -\lambda \right) ^{m}\left( n-l\right) _{m}\left( x\right) _{n-l-m}-K_{n-l,4}\left( x+\lambda -1\mid \lambda \right) \right\} . \end{aligned}$$

Let us consider the following two Sheffer sequences:

2.56 $$\begin{aligned}&K_{n,3}\left( x\mid \lambda \right) \sim \left( \frac{\lambda t}{\log \left( 1+\lambda \left( e^{t}-1\right) \right) },e^{t}-1\right) ,\quad \left( x\right) ^{\left( n\right) }=x\left( x+1\right) \cdots \left( x+\left( n-1\right) \right) \sim \left( 1,1-e^{-t}\right) . \end{aligned}$$

From (1.14) and (1.15), we have

2.57 $$\begin{aligned} K_{n,3}\left( x\mid \lambda \right) =\sum _{m=0}^{n}C_{n,m}\left( x\right) ^{\left( m\right) }, \end{aligned}$$

where

2.58 $$\begin{aligned}&C_{n,m}\nonumber \\&=\frac{1}{m!}\left\langle \left. \frac{\log \left( 1+\lambda t\right) }{\lambda \log \left( 1+ t\right) }\left( 1-\frac{1}{1+t}\right) ^{m}\right| x^{n}\right\rangle \nonumber \\&=\frac{1}{m!}\left\langle \left. \frac{\log \left( 1+\lambda t\right) }{\lambda \log \left( 1+ t\right) }\right| \sum _{l=0}^{\infty }\left( -1\right) ^{l}\left( m+l-1\right) _{l}\frac{t^{m+l}}{l!}x^{n}\right\rangle \nonumber \\&=\frac{1}{m!}\sum _{l=0}^{n-m}\left( -1\right) ^{l}\left( m+l-1\right) _{l}\frac{\left( n\right) _{m+l}}{l!}\left\langle \left. \frac{\log \left( 1+\lambda t\right) }{\lambda t}\right| \frac{t}{\log \left( 1+t\right) }x^{n-m-l}\right\rangle \nonumber \\&=\frac{1}{m!}\sum _{l=0}^{n-m}\left( -1\right) ^{l}\left( m+l-1\right) _{l}\frac{\left( n\right) _{m+l}}{l!}\left\langle \left. \frac{\log \left( 1+\lambda t\right) }{\lambda t}\right| \sum _{k=0}^{\infty }b_{k}\frac{t^{k}}{k!}x^{n-m-l}\right\rangle \nonumber \\&=\sum _{l=0}^{n-m}\left( -1\right) ^{l}l!\left( {\begin{array}{c}m+l-1\\ l\end{array}}\right) \left( {\begin{array}{c}n\\ m+l\end{array}}\right) \left( {\begin{array}{c}m+l\\ m\end{array}}\right) \nonumber \\&\quad \times \sum _{k=0}^{n-m-l}\left( {\begin{array}{c}n-m-l\\ k\end{array}}\right) b_{k}\left\langle \left. \frac{\log \left( 1+\lambda t\right) }{\lambda t}\right| x^{n-m-l-k}\right\rangle \nonumber \\&=\sum _{l=0}^{n-m}\sum _{k=0}^{n-m-l}\left( -1\right) ^{l}l!\left( {\begin{array}{c}m+l-1\\ l\end{array}}\right) \left( {\begin{array}{c}n\\ m+l\end{array}}\right) \left( {\begin{array}{c}m+l\\ m\end{array}}\right) \nonumber \\&\quad \times \left( {\begin{array}{c}n-m-l\\ k\end{array}}\right) b_{k}D_{n-m-l-k}\lambda ^{n-m-l-k}\nonumber \\&=\sum _{l=0}^{n-m}\sum _{k=0}^{n-m-l}\left( -1\right) ^{l}l!\left( {\begin{array}{c}m+l-1\\ l\end{array}}\right) \left( {\begin{array}{c}n\\ m+l\end{array}}\right) \left( {\begin{array}{c}m+l\\ m\end{array}}\right) \nonumber \\&\quad \times \left( {\begin{array}{c}n-m-l\\ k\end{array}}\right) b_{n-m-l-k}D_{k}\lambda ^{k} \end{aligned}$$

Therefore, by (2.57) and (2.58), we obtain the following theorem.

### **Theorem 11**

*For *$n\ge 0$*, we have*

$$\begin{aligned}&K_{n,3}\left( x\mid \lambda \right) \\&=\sum _{m=0}^{n}\left\{ \sum _{l=0}^{n-m}\sum _{k=0}^{n-m-l}\left( -1\right) ^{l}l!\left( {\begin{array}{c}m+l-1\\ l\end{array}}\right) \left( {\begin{array}{c}n\\ m+l\end{array}}\right) \right. \\&\quad \times \left. \left( {\begin{array}{c}m+l\\ m\end{array}}\right) \left( {\begin{array}{c}n-m-l\\ k\end{array}}\right) b_{n-m-l-k}D_{k}\lambda ^{k}\right\} \left( x\right) ^{\left( m\right) }. \end{aligned}$$

For $K_{n,4}\left( x\mid \lambda \right) \sim \left( \frac{e^{\lambda t}-1}{\log \left( 1+\lambda \left( e^{t}-1\right) \right) },e^{t}-1\right)$, $\left( x\right) ^{\left( n\right) }\sim \left( 1,1-e^{-t}\right) ,$ we have

2.59 $$\begin{aligned} K_{n,4}\left( x\mid \lambda \right) =\sum _{m=0}^{n}C_{n,m}\left( x\right) ^{\left( m\right) }, \end{aligned}$$

where

2.60 $$\begin{aligned}&C_{n,m}\nonumber \\&=\frac{1}{m!}\left\langle \left. \frac{\log \left( 1+\lambda t\right) }{\left( 1+t\right) ^{\lambda }-1}\left( 1-\frac{1}{1+t}\right) ^{m}\right| x^{n}\right\rangle \nonumber \\&=\frac{1}{m!}\left\langle \left. \frac{\log \left( 1+\lambda t\right) }{\left( 1+t\right) ^{\lambda }-1}\right| \sum _{l=0}^{\infty }\left( -1\right) ^{l}\left( m+l-1\right) _{l}\frac{t^{m+l}}{l!}x^{n}\right\rangle \nonumber \\&=\frac{1}{m!}\sum _{l=0}^{n-m}\left( -1\right) ^{l}\left( m+l-1\right) _{l}\frac{\left( n\right) _{m+l}}{l!}\left\langle \left. \frac{\log \left( 1+\lambda t\right) }{\lambda t}\right| \frac{\lambda t}{\left( 1+t\right) ^{\lambda }-1}x^{n-m-l}\right\rangle \nonumber \\&=\frac{1}{m!}\sum _{l=0}^{n-m}\left( -1\right) ^{l}\left( m+l-1\right) _{l}\frac{\left( n\right) _{m+l}}{l!}\left\langle \left. \frac{\log \left( 1+\lambda t\right) }{\lambda t}\right| \sum _{k=0}^{\infty }K_{k}\left( \lambda \right) \frac{t^{k}}{k!}x^{n-m-l}\right\rangle \nonumber \\&=\frac{1}{m!}\sum _{l=0}^{n-m}\left( -1\right) ^{l}\left( m+l-1\right) _{l}\frac{\left( n\right) _{m+l}}{l!}\sum _{k=0}^{n-m-l}\left( {\begin{array}{c}n-m-l\\ k\end{array}}\right) K_{k}\left( \lambda \right) \nonumber \\&\quad \times \left\langle \left. \frac{\log \left( 1+\lambda t\right) }{\lambda t}\right| x^{n-m-l-k}\right\rangle \nonumber \\&=\frac{1}{m!}\sum _{l=0}^{n-m}\left( -1\right) ^{l}\left( m+l-1\right) _{l}\frac{\left( n\right) _{m+l}}{l!}\nonumber \\&\quad \times \sum _{k=0}^{n-m-l}\left( {\begin{array}{c}n-m-l\\ k\end{array}}\right) K_{k}\left( \lambda \right) D_{n-m-l-k}\lambda ^{n-m-l-k}\nonumber \\&=\sum _{l=0}^{n-m}\sum _{k=0}^{n-m-l}\left( -1\right) ^{l}l!\left( {\begin{array}{c}m+l-1\\ l\end{array}}\right) \left( {\begin{array}{c}n\\ m+l\end{array}}\right) \left( {\begin{array}{c}m+l\\ m\end{array}}\right) \nonumber \\&\quad \times \left( {\begin{array}{c}n-m-l\\ k\end{array}}\right) K_{n-m-l-k}\left( \lambda \right) D_{k}\lambda ^{k}. \end{aligned}$$

Therefore, by (2.59) and (2.60), we obtain the following theorem.

### **Theorem 12**

*For*$n\ge 0$*, we have*

$$\begin{aligned}&K_{n,4}\left( x\mid \lambda \right) \\&=\sum _{m=0}^{n}\left\{ \sum _{l=0}^{n-m}\sum _{k=0}^{n-m-l}\left( -1\right) ^{l}l!\left( {\begin{array}{c}m+l-1\\ l\end{array}}\right) \right. \\&\quad \times \left. \left( {\begin{array}{c}n\\ m+l\end{array}}\right) \left( {\begin{array}{c}m+l\\ m\end{array}}\right) \left( {\begin{array}{c}n-m-l\\ k\end{array}}\right) K_{n-m-l-k}\left( \lambda \right) D_{k}\lambda ^{k}\right\} \left( x\right) ^{\left( m\right) }. \end{aligned}$$

Let us consider the following two Sheffer sequences:

$$\begin{aligned} K_{n,3}\left( x\mid \lambda \right) \sim \left( \frac{\lambda t}{\log \left( 1+\lambda \left( e^{t}-1\right) \right) },e^{t}-1\right) ,\quad B_{n}^{\left( s\right) }\left( x\right) \sim \left( \left( \frac{e^{t}-1}{t}\right) ^{s},t\right) . \end{aligned}$$

Note that

$$\begin{aligned} \sum _{n=0}^{\infty }B_{n}^{\left( s\right) }\left( x\right) =\left( \frac{t}{e^{t}-1}\right) ^{s}e^{xt},\quad \left( \text {see Kim et al. 2015; Sen et al. 2013; Ustinov et al. 2002 }\right) , \end{aligned}$$

where $B_{n}^{\left( s\right) }\left( x\right)$ are called the higher-order Bernoulli polynomials.

From (1.14) and (1.15), we have

2.61 $$\begin{aligned} K_{n,3}\left( x\mid \lambda \right) =\sum _{m=0}^{n}C_{n,m}B_{m}^{\left( s\right) }\left( x\right) , \end{aligned}$$

where

2.62 $$\begin{aligned}&C_{n,m}\nonumber \\&=\frac{1}{m!}\left\langle \left. \left( \frac{t}{\log \left( 1+t\right) }\right) ^{s}\frac{\log \left( 1+\lambda t\right) }{\lambda \log \left( 1+t\right) }\left( \log \left( 1+t\right) \right) ^{m}\right| x^{n}\right\rangle \nonumber \\&=\left\langle \left. \left( \frac{t}{\log \left( 1+t\right) }\right) ^{s}\frac{\log \left( 1+\lambda t\right) }{\lambda \log \left( 1+t\right) }\right| \frac{1}{m!}\left( \log \left( 1+t\right) \right) ^{m}x^{n}\right\rangle \nonumber \\&=\left\langle \left. \left( \frac{t}{\log \left( 1+t\right) }\right) ^{s}\frac{\log \left( 1+\lambda t\right) }{\lambda \log \left( 1+t\right) }\right| \sum _{l=m}^{\infty }S_{1}\left( l,m\right) \frac{t^{l}}{l!}x^{n}\right\rangle \nonumber \\&=\sum _{l=m}^{n}\left( {\begin{array}{c}n\\ l\end{array}}\right) S_{1}\left( l,m\right) \left\langle \left. \left( \frac{t}{\log \left( 1+t\right) }\right) ^{s}\right| \frac{\log \left( 1+\lambda t\right) }{\lambda \log \left( 1+t\right) }x^{n-l}\right\rangle \nonumber \\&=\sum _{l=m}^{n}\left( {\begin{array}{c}n\\ l\end{array}}\right) S_{1}\left( l,m\right) \left\langle \left. \left( \frac{t}{\log \left( 1+t\right) }\right) ^{s}\right| \sum _{k=0}^{\infty }K_{k,3}\left( \lambda \right) \frac{t^{k}}{k!}x^{n-l}\right\rangle \nonumber \\&=\sum _{l=m}^{n}\left( {\begin{array}{c}n\\ l\end{array}}\right) S_{1}\left( l,m\right) \sum _{k=0}^{n-l}\left( {\begin{array}{c}n-l\\ k\end{array}}\right) K_{k,3}\left( \lambda \right) \left\langle \left. \left( \frac{t}{\log \left( 1+t\right) }\right) ^{s}\right| x^{n-l-k}\right\rangle \nonumber \\&=\sum _{l=m}^{n}\left( {\begin{array}{c}n\\ l\end{array}}\right) S_{1}\left( l,m\right) \sum _{k=0}^{n-l}\left( {\begin{array}{c}n-l\\ k\end{array}}\right) K_{k,3}\left( \lambda \right) b_{n-l-k}^{\left( s\right) }\nonumber \\&=\sum _{l=m}^{n}\sum _{k=0}^{n-l}\left( {\begin{array}{c}n\\ l\end{array}}\right) \left( {\begin{array}{c}n-l\\ k\end{array}}\right) S_{1}\left( l,m\right) K_{k,3}\left( \lambda \right) b_{n-l-k}^{\left( s\right) }. \end{aligned}$$

Here, the Bernoulli numbers of the second kind of order *s* are defined by the generating function

$$\begin{aligned} \left( \frac{t}{\log \left( 1+t\right) }\right) ^{s}=\sum _{j=0}^{\infty }b_{j}^{\left( s\right) }\frac{t^{j}}{j!},\quad \left( \text {see Kim et al. 2015; Roman 1984}\right) . \end{aligned}$$

Therefore, by (2.61) and (2.62), we obtain the following theorem.

### **Theorem 13**

*For*$n\ge 0$*, we have*

$$\begin{aligned} K_{n,3}\left( x\mid \lambda \right) =\sum _{m=0}^{n}\left( \sum _{l=m}^{n}\sum _{k=0}^{n-l}\left( {\begin{array}{c}n\\ l\end{array}}\right) \left( {\begin{array}{c}n-l\\ k\end{array}}\right) S_{1}\left( l,m\right) K_{k,3}\left( \lambda \right) b_{n-l-k}^{\left( s\right) }\right) B_{m}^{\left( s\right) }\left( x\right) . \end{aligned}$$

### *Remark*

In a similar manner, one shows that, for $n\ge 0$,

$$\begin{aligned} K_{n,4}\left( x\mid \lambda \right) =\sum _{m=0}^{n}\left( \sum _{l=m}^{n}\sum _{k=0}^{n-l}\left( {\begin{array}{c}n\\ l\end{array}}\right) \left( {\begin{array}{c}n-l\\ k\end{array}}\right) S_{1}\left( l,m\right) K_{k,4}\left( \lambda \right) b_{n-l-k}^{\left( s\right) }\right) B_{m}^{\left( s\right) }\left( x\right) . \end{aligned}$$

For $\mu \in \mathbb {C}$ with $\mu \ne 1$, $s\in \mathbb {N}$, the Frobenius-Euler polynomials of order *s* are defined by the generating function

$$\begin{aligned} \left( \frac{1-\mu }{e^{t}-\mu }\right) ^{s}e^{xt}=\sum _{n=0}^{\infty }H_{n}^{\left( s\right) }\left( x\mid \mu \right) \frac{t^{n}}{n!},\quad \left( \text {see [1,16]}\right) . \end{aligned}$$

For $K_{n,3}\left( x\mid \lambda \right) \sim \left( \frac{\lambda t}{\log \left( 1+\lambda \left( e^{t}-1\right) \right) },e^{t}-1\right)$, $H_{n}^{\left( s\right) }\left( x\mid \mu \right) \sim \left( \left( \frac{e^{t}-\mu }{1-\mu }\right) ^{s},t\right)$, we have

2.63 $$\begin{aligned} K_{n,3}\left( x\mid \lambda \right) =\sum _{m=0}^{n}C_{n,m}H_{m}^{\left( s\right) }\left( x\mid \mu \right) , \end{aligned}$$

where

2.64 $$\begin{aligned} C_{n,m}&=\frac{1}{m!}\left\langle \left. \left( \frac{1-\mu +t}{1-\mu }\right) ^{s}\frac{\log \left( 1+\lambda t\right) }{\lambda \log \left( 1+t\right) }\left( \log \left( 1+t\right) \right) ^{m}\right| x^{n}\right\rangle \nonumber \\&=\left\langle \left. \left( \frac{1-\mu +t}{1-\mu }\right) ^{s}\frac{\log \left( 1+\lambda t\right) }{\lambda \log \left( 1+t\right) }\right| \frac{1}{m!}\left( \log \left( 1+t\right) \right) ^{m}x^{n}\right\rangle \nonumber \\&=\left\langle \left. \left( \frac{1-\mu +t}{1-\mu }\right) ^{s}\frac{\log \left( 1+\lambda t\right) }{\lambda \log \left( 1+t\right) }\right| \sum _{l=m}^{\infty }S_{1}\left( l,m\right) \frac{t^{l}}{l!}x^{n}\right\rangle \nonumber \\&=\sum _{l=m}^{n}\left( {\begin{array}{c}n\\ l\end{array}}\right) S_{1}\left( l,m\right) \left\langle \left. \left( \frac{1-\mu +t}{1-\mu }\right) ^{s}\right| \frac{\log \left( 1+\lambda t\right) }{\lambda \log \left( 1+t\right) }x^{n-l}\right\rangle \nonumber \\&=\sum _{l=m}^{n}\left( {\begin{array}{c}n\\ l\end{array}}\right) S_{1}\left( l,m\right) \left\langle \left. \left( \frac{1-\mu +t}{1-\mu }\right) ^{s}\right| \sum _{k=0}^{\infty }K_{k,3}\left( \lambda \right) \frac{t^{k}}{k!}x^{n-l}\right\rangle \nonumber \\&=\sum _{l=m}^{n}\left( {\begin{array}{c}n\\ l\end{array}}\right) S_{1}\left( l,m\right) \sum _{k=0}^{n-l}\left( {\begin{array}{c}n-l\\ k\end{array}}\right) K_{k,3}\left( \lambda \right) \left\langle \left. \left( \frac{1-\mu +t}{1-\mu }\right) ^{s}\right| x^{n-l-k}\right\rangle \nonumber \\&=\frac{1}{\left( 1-\mu \right) ^{s}}\sum _{l=m}^{n}\left( {\begin{array}{c}n\\ l\end{array}}\right) S_{1}\left( l,m\right) \sum _{k=0}^{n-l}\left( {\begin{array}{c}n-l\\ k\end{array}}\right) K_{k,3}\left( \lambda \right) \nonumber \\&\quad \times \sum _{j=0}^{s}\left( {\begin{array}{c}s\\ j\end{array}}\right) \left( 1-\mu \right) ^{s-j}\left\langle \left. t^{j}\right| x^{n-l-k}\right\rangle \nonumber \\&=\frac{1}{\left( 1-\mu \right) ^{s}}\sum _{l=m}^{n}\left( {\begin{array}{c}n\\ l\end{array}}\right) S_{1}\left( l,m\right) \sum _{k=0}^{n-l}\left( {\begin{array}{c}n-l\\ k\end{array}}\right) K_{n-l-k,3}\left( \lambda \right) \nonumber \\&\quad \times \sum _{j=0}^{s}\left( {\begin{array}{c}s\\ j\end{array}}\right) \left( 1-\mu \right) ^{s-j}\left\langle \left. t^{j}\right| x^{k}\right\rangle \nonumber \\&=\frac{1}{\left( 1-\mu \right) ^{s}}\sum _{l=m}^{n}\left( {\begin{array}{c}n\\ l\end{array}}\right) S_{1}\left( l,m\right) \sum _{k=0}^{n-l}\left( {\begin{array}{c}n-l\\ k\end{array}}\right) K_{n-l-k,3}\left( \lambda \right) k!\left( {\begin{array}{c}s\\ k\end{array}}\right) \left( 1-\mu \right) ^{s-k}\nonumber \\&=\sum _{l=m}^{n}\sum _{k=0}^{n-l}\left( {\begin{array}{c}n\\ l\end{array}}\right) \left( {\begin{array}{c}n-l\\ k\end{array}}\right) \left( {\begin{array}{c}s\\ k\end{array}}\right) \frac{k!}{\left( 1-\mu \right) ^{k}}S_{1}\left( l,m\right) K_{n-l-k,3}\left( \lambda \right) . \end{aligned}$$

Therefore, by (2.63) and (2.64), we obtain the following theorem.

### **Theorem 14**

*For*$n\ge 0$*, we have*

$$\begin{aligned}&K_{n,3}\left( x\mid \lambda \right) \\&=\sum _{m=0}^{n}\left( \sum _{l=m}^{n}\sum _{k=0}^{n-l}\left( {\begin{array}{c}n\\ l\end{array}}\right) \left( {\begin{array}{c}n-l\\ k\end{array}}\right) \left( {\begin{array}{c}s\\ k\end{array}}\right) \frac{k!}{\left( 1-\mu \right) ^{k}}S_{1}\left( l,m\right) K_{n-l-k,3}\left( \lambda \right) \right) H_{m}^{\left( s\right) }\left( x\mid \mu \right) . \end{aligned}$$

### *Remark*

Proceeding similarly to the above, one can show that, for $n\ge 0$,

$$\begin{aligned}&K_{n,4}\left( x\mid \lambda \right) \\&=\sum _{m=0}^{n}\left( \sum _{l=m}^{n}\sum _{k=0}^{n-l}\left( {\begin{array}{c}n\\ l\end{array}}\right) \left( {\begin{array}{c}n-l\\ k\end{array}}\right) \left( {\begin{array}{c}s\\ k\end{array}}\right) \frac{k!}{\left( 1-\mu \right) ^{k}}S_{1}\left( l,m\right) K_{n-l-k,4}\left( \lambda \right) \right) H_{m}^{\left( s\right) }\left( x\mid \mu \right) . \end{aligned}$$

## Conclusion

The first degenerate version of the Bernoulli polynomials of the second kind appeared in the paper by Korobov (1996, 2001). Here, we study two degenerate versions of the Bernoulli polynomials of the second kind which will be called Korobov polynomials of third kind and of the fourth kind. Some properties, identities, recurrence relations and connections with other polynomials are investigated by using umbral calculus.
